# Neuroinflammation in perioperative neurocognitive disorders: From bench to the bedside

**DOI:** 10.1111/cns.13794

**Published:** 2022-01-06

**Authors:** Yang Liu, Huiqun Fu, Tianlong Wang

**Affiliations:** ^1^ Department of Anesthesiology Xuanwu Hospital Capital Medical University Beijing China

**Keywords:** astrocyte, cognitive impairment, microglia, neuroinflammation, perioperative neurocognitive disorders

## Abstract

The perioperative neurocognitive disorders (PNDs) are one of the most common complications in elderly patients characterized by various forms of cognitive decline after anesthesia and surgery. Although the etiology for PNDs remained unclear, neuroinflammation has been characterized as one of the major causes, especially in the elderly patients. The activation of glial cells including microglia and astrocytes plays a significant role in the inflammatory responses in central nerve system (CNS). Although carefully designed, clinical studies on PNDs showed controversial results. Meanwhile, preclinical studies provided evidence from various levels, including behavior performance, protein levels, and gene expression. In this review, we summarize high‐quality studies and recent advances from both clinical and preclinical studies and provide a broad view from the onset of PNDs to its potential therapeutic targets. Future studies are needed to investigate the signaling pathways in PNDs for prevention and treatment, as well as the relationship of PNDs and future neurocognitive dysfunction.

## Graphical abstract

In this review, we focused on the neuroinflammation in perioperative neurocognitive disorders (PNDs) from bench to bedside. The review not only presented the latest findings of PNDs in both clinical practice and preclinical experiment, but also pointed out useful biomarkers and potential therapeutic strategies.

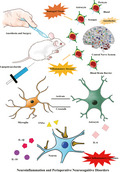

## 1 INTRODUCTION

Throughout the ages, general anesthesia has been recognized as an “instantly reversible condition” that leaves no sequalae after emergence, despite remarkable alterations in consciousness and similarly dramatic changes in other organ systems.^1^ However, increasing amount of evidence has shown that general anesthesia is not simply an “instantly reversible condition,” but has acute, even long‐lasting influence on central nerve system (CNS).^2^, ^3^, ^4^ Coexisting with general anesthesia, orthopedic or cardiac surgeries often lead to acute or long‐term cognitive decline after surgical procedures.^5^ These phenomena urge anesthetists to pay close attention to neurocognitive outcome in clinical practice.

Traditionally, all forms of cognitive impairments after anesthesia and surgery were termed postoperative cognitive dysfunction (POCD), which was recently recommended to change into perioperative neurocognitive disorder (PND).^6^, ^7^ Depending on the time of onset, PNDs can be classified into neurocognitive disorder (occurring before anesthesia and surgery), postoperative delirium (POD, delirium occurring within hours to days after surgery), delayed neurocognitive recovery (occurring up to 30 days after anesthesia and surgery), and postoperative neurocognitive disorder (POND, occurring within weeks to months after anesthesia and surgery).^7^ This classification aligns well with the phenotypically similar diseases defined in the Diagnostic and Statistical Manual of Mental Disorders, version 5 (DSM‐5) and International Classification of Disease‐10 (ICD‐10).^8^, ^9^ In order to distinguish PNDs and traditional dementia (such as Alzheimer's disease), the use of DSM‐5 is also needed.^10^ Since the definition for cognitive impairment after anesthesia and surgery has changed over the past several years and different cognitive evaluation tests were used in different studies (eg, whether the learning effect was corrected, and which neuropsychological test was used),^11^, ^12^ the exact rate of PNDs remain unclear. Nevertheless, consensus is made that newly diagnosed cognitive dysfunction after anesthesia and surgery may increase the risk of future brain dysfunction, including dementia,^10^, ^13^ Alzheimer's disease (AD),^14^, ^15^ and long‐term cognitive decline,^4^, ^16^ making PNDs a highly concerning condition, especially in the elderly population. In this way, the PNDs in this review is also focused postoperatively.

Neuroinflammation caused by anesthesia exposure and surgery has been characterized as a major contributor to cognitive decline in PNDs.^17^ Recent studies reported that systemic stressors and other inflammatory cytokines broke down the blood brain barrier (BBB) and further resulted in neuroinflammation.^17^, ^18^, ^19^ Preclinical experiments demonstrated that inflammatory cytokines secreted by either microglia or astrocytes resulted in cognitive decline,^20^, ^21^ impaired synaptic plasticity,^22^, ^23^ and brain functional changes.^20^ Clinical data showed that PND patients had increased peripheral inflammatory cytokine levels,^24^ increased risks of future cognitive impairment^25^ and brain function decline.^26^ Although effort has been made both clinically and preclinically to investigate the mechanisms, risk factors, clinical onset and development, and the long‐term outcome of PNDs, effective approaches to reverse this neurocognitive situation are lacking. What is more, the detailed mechanisms underlying the complicated clinical symptoms are still unclear and need further investigation.

In this article, we present a broad review of literatures regarding neuroinflammation in PNDs and its translational potential. Both clinical and preclinical studies are reviewed to provide an in‐depth understanding of the molecular mechanisms on the perioperative neuroinflammation and the pathogenesis of PNDs. We also summarize the potential targets and treatment strategies to manage this neurocognitive status, in order for a better long‐term outcome after anesthesia and surgery in the elderly.

## 2 CURRENT UNDERSTANDING IN PNDS: WHAT WE ALREADY KNOW, AND WE WHAT DO NOT KNOW YET?

Most of clinical studies focused on the POD, delayed neurocognitive recovery, and POND (based on the new definition according to the time of onset). Carefully designed clinical studies proved that intraoperative anesthesia depth monitoring,^27^, ^28^, ^29^ dexmedetomidine infusion^30^, ^31^ intravenous anesthesia (as compared with inhalational anesthesia),^32^ and multi‐modal analgesia^25^, ^33^, ^34^ are effective to reduce the incidence of PNDs. Other approaches such as management of intraoperative body temperature^35^, ^36^ and blood pressure^37^ may also help (Table 1). However, clinical study results differ from one another. For example, some studies showed that intraoperative blood pressure,^38^ dexmedetomidine,^39^ and anesthesia method^40^, ^41^ may not be as effective in reducing the incidence of PNDs as expected (Table 1). The reasons for these discrepancies may be the followings: (1) the mechanisms for PNDs may vary from case to case, although they share similar clinical symptoms. Variations in the time‐frame of inflammatory cytokines after abdominal and cardiac surgeries have been observed in preclinical studies^42^; (2) the definition for postoperative cognitive decline has been changing over the past years,^17^ making it hard to evaluate the a certain factor across multiple studies.

**TABLE 1:** Studies concerning perioperative neurocognitive disorders (PNDs)

References	Type of study	Method	Type of PNDs	Main findings	Conclusions
MacKenzie et al (2018)	Meta‐analysis	Anesthesia depth	POD	POD (38.0% reduction)	Electroencephalogram‐guided anesthesia is associated with decreased POD
Bocskai et al (2020)	Meta‐analysis	Anesthesia depth	POD POND	POD (6.7% reduction) PND (3.0% reduction)	BIS‐guided anesthesia reduced rate of POD at 1 day and PND at 12 weeks after anesthesia and surgery
Yang et al (2021)	RCT	Anesthesia depth	POD	MoCA score (average 1.24 higher, first 7 days)	Multi‐modal brain monitoring improves postoperative neurocognition.
Zhao et al (2020)	RCT	Dexmedetomidine	POD Delayed Neurocognitive Recovery	POD (decreased on day 1–3, *p *< 0.05) Delayed neurocognitive recovery (decreased on day 7, *p *< 0.05)	Intraoperative use of dexmedetomidine significantly attenuated the rate of POD and delayed neurocognitive recovery
Su, et al, 2016	RCT	Dexmedetomidine	POD	POD (14.0% reduction, first 7 days)	Use of dexmedetomidine decreases the incidence of POD in ICU in patients >65 yrs undergoing non‐cardiac surgery.
Deiner et al (2017)	RCT	Dexmedetomidine	POD	POD (increased for 0.8%, *p *> 0.94)	Use of dexmedetomidine cannot prevent POD from happening.
Zhang et al (2018)	RCT	Intravenous anesthesia	Delayed Neurocognitive Recovery	Delayed neurocognitive recovery (8.4% reduction, first 7 days)	Propofol reduced the rate of delayed neurocognitive recovery as compared with sevoflurane.
Konishi et al (2018)	RCT	Intravenous anesthesia	Delayed Neurocognitive Recovery POND	Delayed neurocognitive recovery (*p *= 0.26) PND (*p *= 0.61 and 0.23, 3 and 12 months after surgery)	No difference was found between propofol and sevoflurane for inducing cognitive impairment
Sun et al (2019)	Meta‐analysis	Intravenous anesthesia	POD	MMSE score (significantly lower in patients using propofol until 7 days)	Propofol had great adverse effect as compared with sevoflurane
Kristek et al (2019)	RCT	Multi‐modal analgesia	POD	POD (22.0% reduction, first 72 hours)	Multi‐modal analgesia significantly reduced the rate of POD.
Subramaniam et al (2019)	RCT	Multi‐modal analgesia	POD	POD length (1 day reduced, first 48 hours)	Acetaminophen reduced the length of POD in elderly patients
Mu et al (2017)	RCT	Multi‐modal analgesia	POD	POD (4.8% reduction, first 5 days)	Multi‐dose of parecoxib supplemented to intravenous morphine decreased the rate of POD without increasing side effects.
Rudiger et al (2016)	Observational	Temperature	POD	Hypothermia (34.5℃ *vs*. 35.1℃)	Low body temperature is one of the major risks for POD in ICU.
Wagner et al (2021)	Retrospective Exploratory	Temperature	POD	Hypothermia (χ^2^ = 54.94, *df* = 4)	A significant relationship was found between hypothermia and POD
Maheshwari et al (2020)	Observational	Blood Pressure	POD	Hypotension (*p *= 0.009, 95% CI: 1.03–1.20)	Intraoperative hypotension is moderately associated with POD within 5 days after surgery.
Feng, et al (2020)	Meta‐analysis	Blood Pressure	POD POND	Hypotension (*p *= 0.10 for POD: *p *= 0.37 for POCD)	No significant correlations between intraoperative hypotension and POD / PND.

Note

The type of PNDs were adjusted according to the latest diagnostic criteria.

Abbreviations: ICU, intensive care unit; MoCA, Montreal Cognitive Assessment; POD, postoperative delirium; POND, postoperative neurocognitive disorder; RCT, randomized controlled trial.

As for the different anesthesia methods, several studies demonstrated that combined anesthesia (eg, combined general anesthesia and epidural anesthesia) or regional anesthesia had lower incidence of PNDs as compared with general anesthesia alone.^43^, ^44^, ^45^ However, there are also studies claiming no significant difference in PND rates between regional and general anesthesia.^46^, ^47^ The contradictory findings in clinical practice pointed out that the mechanism for PNDs may vary, suggesting that mechanistic studies are obviously warranted.

While clinical studies reported controversial results, most of the preclinical experiments claimed that the neuroinflammation triggered by peripheral inflammatory cytokines/stressors is the major cause of clinical symptoms.^17^, ^18^, ^48^ Previous studies found that surgical trauma‐induced systemic inflammation may cause neuroinflammation through damage‐associated molecular patterns (DAMPs), complement cascade and coagulation cascade, all of which could further induce glial activation in the CNS.^18^ Inflammatory responses triggered by surgery and anesthesia broke down the blood brain barrier (BBB) and cause further neuroinflammation,^18^ the process of which started the inflammatory response in CNS. In elderly patients, the progressive loss of hippocampal BBB integrity has already been proved in individuals without evident cognitive impairments.^49^ Preclinical experiment (LPS stimulation) shown that the microglia and astrocyte activation and inflammatory cytokines expression caused long‐lasting cognitive impairment in rodent animals.^20^, ^23^, ^50^ Meanwhile, the anesthetic agents also induced significant microglia/astrocyte activation directly in CNS.^51^, ^52^ After the microglia and astrocyte activation, both types of the glia secreted inflammatory cytokines including interleukin (IL)‐1β, tumor necrosis factor (TNF) α, and other cognitive‐related cytokines, in the progress of neuroinflammation,^20^, ^53^ causing impaired synaptic plasticity,^54^ which further induced cognitive dysfunction.

Although there are various studies demonstrating the crosstalk between microglia and astrocyte, even the neurons in cognitive dysfunction, only few studies focused on the effect of PNDs. In this case, the effect of the glia response behind the PNDs pathologic process should be further studied to investigate the mechanism behind the clinical symptoms.

## 3 GLIAL ACTIVATION: THE SOURCE OF NEUROINFLAMMATION AND THE TARGET OF CLINICAL TRANSLATION

Although details remain unclear, increasing body of evidence supports the idea that both the microglia and astrocytes, and their interactions, contribute to the neuroinflammatory processes.^55^, ^56^, ^57^ Long‐term activation of microglia and astrocytes result in long‐term synaptic inhibition and cognitive dysfunction,^17^, ^23^ inflammatory responses in the hippocampus,^17^, ^18^ and eventually neurodegenerative diseases.^58^, ^59^

### 3.1 Microglia activation

Microglia, one of the major resident cells in CNS (accounting for about 5–10% of the total cells in human and mice), are traditionally considered the major source of neuroinflammatory response.^60^ They have long been believed to be CNS‐resident phagocytes to remove excessive debris functionally. In recent years, by means of sequencing technologies and other advancing methods, it was revealed that microglia are not just passive bystanders of CNS pathologies, but also determinants of diseases.^61^, ^62^

**FIGURE 1:**
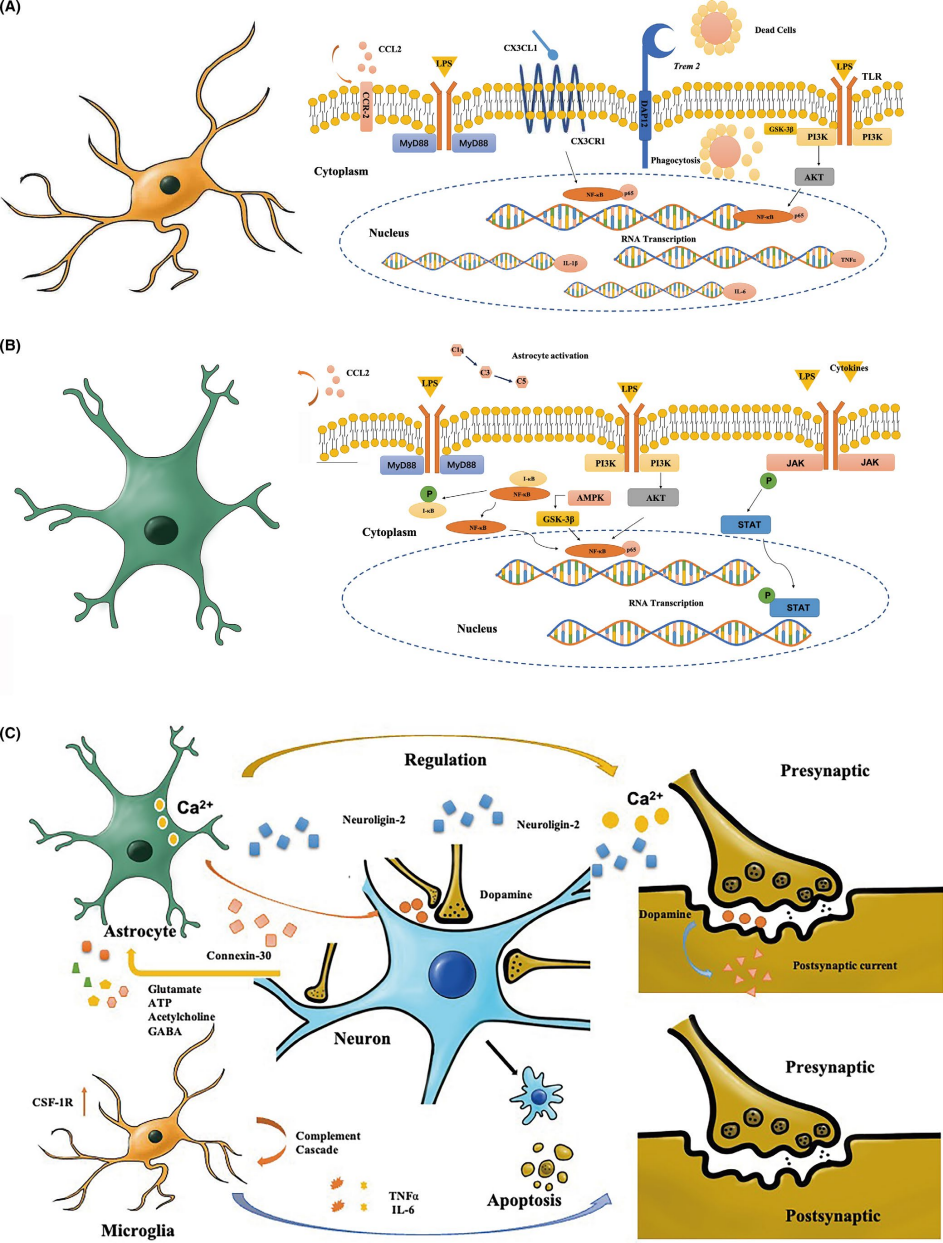
Activated microglia and activated astrocytes in neuroinflammation. (A) Representative image of activated microglia in neuroinflammation. (B) Representative image of activated astrocytes in neuroinflammation. (C) Representative image of activated astrocytes and microglia on neuron dysfunction. CX3CL1/CX3CR1, chemokine fractalkine ligand 1/chemokine fractalkine receptor; LPS, lipopolysaccharide; IL‐1β, interleukin‐1 beta; MyD88, myeloid differentiation factor 88; NF‐κB, nuclear factor‐kappa B; TNFα, tumor necrosis factor alpha; PI3K, phosphoinositide 3‐kinase; AKT, Ser/Thr kinase; JAK, Janus kinase; STAT, signal transducer and activator of transcription; I‐ κB, inhibitor of NF‐κB; P, Phosphate; AMPK, adenosine 5’‐monophosphate‐activated protein kinase; GSK‐3β, glycogen synthase kinase 3β; *Trem*, triggering receptor expressed on myeloid cell

Anesthesia and surgery‐induced microglial activation has been demonstrated to cause cognitive dysfunction (Figure 1A). Preclinical study has shown that the TLR/GSK‐3β/PI3K/AKT signaling pathway reduced the activation of the nuclear factor‐kappa B (NF‐κB) signaling pathway in microglia, decreased the M1 type (classical activation) and increased the M2 type (alternating activation) transformation^63^ in a PND model of tibial fracture surgery. In another PND model upon exposure of inhalational anesthesia (3% sevoflurane) in pregnant rats, the NF‐κB signaling pathway played a significant role in microglia activation, which further induced the upregulation of inflammatory cytokines (IL‐1β, IL‐6, and TNFα) and cognitive dysfunction in the descendent pups.^64^ The *Trem* 2, which is mostly seen in activated microglia in AD patients,^65^, ^66^ was also overexpressed in PND preclinical models. Both preclinical (mice with liver lobe resection) and clinical studies (hip‐fracture patients) proved significant changes in *Trem* 2 associated with cognitive impairment.^67^, ^68^ The microglial CX3CL1/CX3CR1 is another overactivated pathway in AD patients, which affects the clearance of Aβ deposits.^69^ Although rarely reported in clinical studies, activation of the microglial CX3CL1/CX3CR1 signaling pathway has been observed in preclinical PND model after tibial fracture.^70^ These results suggested that PNDs and neurodegenerative diseases may share similar pathways. Interestingly, aside from directly activated by stress stimulators, the microglia can also be activated indirectly by astrocytes via the CCL2‐CCR2 signaling pathway.^71^ In the tibial fracture PND model, the upregulation of astrocyte‐derived CCL2 induced the overexpression of microglial CCR2, while blockade of CCR2 by RS504393 attenuated microglial inflammatory responses and improved cognitive function.

Meanwhile, perioperative events including hypoxia (STAT1 protein activation drives M1 activation),^72^ cerebral ischemia (CysLT_2_R‐ERK1/2 pathway mediates M1 polarization),^73^ hemorrhage (activating the PKA/CREB signaling pathway may promote M2 polarization; IL‐15 mediates the crosstalk of astrocyte and microglia in disease development; using EPZ6438 attenuates neuroinflammation by H3k27me3/SOCS3/TRAF6/NF‐κB signaling pathway after hemorrhage),^74^, ^75^, ^76^ Aβ accumulation (promotes M1 polarization),^77^ autoimmune inflammatory disease (P2×4R signaling pathway favored the microglia phagocytosis),^78^ and oxidative stress (reducing oxidative stress also attenuates M1 polarization)^79^ may also cause microglial activation. However, one should note that not all of these signaling pathways contributes to cognitive impairment.

People then asked whether microglia are the only mediator in neuroinflammation. Recent studies showed that microglia also activate astrocytes in PNDs.^80^, ^81^ For example, eliminating early microglial activation in hippocampus attenuated etomidate‐induced long‐term hippocampal astrocyte activation.^82^ This finding suggested that microglia activation occurs at early phase, while subsequent astrocyte may be responsible for long‐term cognitive impairment.

### 3.2 Astrocyte activation

Being one of the most abundant glia cells in the CNS (comprising for at least 50% of the brain and spinal cells by number in human and mice), the major function of the astrocyte is believed to participate the BBB maintenance, modulating synaptic plasticity, as well as neuronal survival and differentiation.^83^, ^84^ However, astrocytes can also be activated by microglia by the complement cascade (C5, C3 and C1q) and further cause neurotoxicity.^85^, ^86^ Thus, the astrocyte activation should also be considered as a significant contributor for neuroinflammation.

Abnormal astrogliosis has been shown to result in PNDs (Figure 1B). The NF‐κB pathway activation triggered by LPS injection or sevoflurane exposure may induce astrocyte activation and cognitive dysfunction in preclinical PND models^64^, ^87^ while administration of pyrrolidine dithiocarbamate (PDTC), an NF‐κB signaling pathway blocker, can improve cognitive behavioral performance.^23^ The PI3K‐AKT signaling pathway may also contribute to astrocytes activation in PNDs.^88^ Preclinical study showed that activating the PI3K‐AKT signaling pathway led to significant complement cascade activation and transformation of astrocytes.^89^ A further study using primary astrocyte culture demonstrated that the STAT3 protein was also involved after the AKT activation.^90^ What is more, the CCL2/CCR2 signaling pathway astrocytes engaged has been proved to increase L‐1β and TNFα secretion in hippocampal CA1 region and in PND rats after tibial fracture surgery.^71^ Oxidative scavenger edaravone or adiponectin have been reported to attenuate cognitive dysfunction and reduce inflammatory cytokines levels (IL‐1β, IL‐6, IL‐18 and TNFα),^91^, ^92^ suggesting that oxidative stress is essential in neuroinflammation. The mechanisms might involve the AMPK/GSK‐3β signaling pathway.

Aside from surgical trauma, perioperative events may also cause abnormal astrogliosis. However, these events are not always related to cognitive dysfunction. Perioperative ischemic/reperfusion events (activated by JAK/STAT3 signaling pathway and other inflammatory cytokines including IL‐6 while modulating by complement activation)^86^, ^93^ and postoperative pain (mainly by NF‐κB, JAK/STAT3, and CXCL13‐CXCR5 signaling pathway).^94^

However, astrocytes also exert a protective effect on cognitive function. Recent study on astrocyte transcriptome sequencing analysis demonstrated that the human astrocytes could promote neuronal survival *in vitro*.^95^ In aged rats undergone abdominal surgery, mesencephalic astrocyte‐derived neurotrophic factor (MANF) was upregulated after surgery, and recombinant human MANF reversed POD‐like behaviors.^96^ This finding may provide new therapeutic targets for PNDs. One should be aware that the astrocyte activation is largely dependent on microglia. The crosstalk between astrocytes and microglia is critical.^56^

### 3.3 Effects of activated microglia and astrocytes on neuronal dysfunction

Large amount of evidence demonstrated that astrocytes release neuroactive substances with a variety of effects on synaptic activity (Figure 1C). Astrocytes regulate neuronal synaptic plasticity by controlling Ca^2+^ flow^97^ and reducing astrocytic Ca^2+^ signaling alters the microcircuits and induces repetitive behavior.^98^ Dopamine in the synaptic cleft could increase astrocytic Ca^2+^ and suppress excitatory postsynaptic currents in mice, which is regulated by Ca^2+^ signaling manifested astrocyte activation.^99^ The astrocytes also express and release several neural factors that affect neuronal functional status. For example, absence of astrocytic neuroligin 2 expression resulted in the loss of cortical excitatory synapse formation,^100^ which may further cause cognitive dysfunction. In contrast, astrocytic connexin 30 may shorten the critical period for visual plasticity in mice during development.^101^ In addition, evidence exists demonstrating neuronal apoptosis induced by astrocytes.^102^

Interestingly, the astrocytes have also been found to respond to neurotransmitters including glutamate, adenosine triphosphate (ATP), acetylcholine, γ‐aminobutyric acid (GABA), etc., in response to neuron dysfunction.^103^, ^104^, ^105^ For example, in N‐methyl‐D‐aspartic acid (NMDA)‐induced excitotoxicity model, the astrocytes can sense neuronal activity^106^ and protects neurons from excessive oxidation injury in by consuming incoming neuronal‐derived fatty acids.^107^

On the contrary, the generation of neurotoxic astrocytes could be induced by activated microglia (Figure 1C).^85^ Non‐activated microglia cultures can hardly induce neurotoxic astrocyte formation, even in the presence of LPS. Microglia also play a significant role in neuronal dysfunction. In mice with cognitive decline and hippocampal synaptic loss, the microglia were shown to activate the complement pathway (mainly C3q).^108^ In tibial fracture, cognitive dysfunction was accompanied by microglia activation and significantly increased secretion of TNFα and IL‐6.^109^ Meanwhile, colony stimulating factor 1 receptor (CSF1R) expression in microglia was upregulated in PND mice (confirmed by impaired performance in Morris water maze) after tibial fracture surgery.^109^ As expected, inhibiting CSF1R in aged mice can improve cognitive function.^110^ Whether activated microglia are a double‐edged sword in neuronal dysfunction needs further investigations.^111^

## 4 CLINICAL BIOMARKERS: IS THERE ANYTHING WE CAN DO TO PREDICT PNDS FROM THE VERY BEGINNING?

Since PNDs are hard to cure, biomarkers would be of great use for preventive purposes. Recent studies reported several available biomarkers, which can be divided into the following three categories: trauma/surgery‐induced inflammatory markers, neurodegeneration disease‐related biomarkers, and neuroimaging markers (Table 2).

**TABLE 2:** Clinical biomarkers for perioperative neurocognitive disorders (PNDs)

Reference	Biomarker	Type	Sample	Evidence	Main findings
Quan et al (2019)	IL‐1β	Inflammatory	Plasma	Clinical	Lower IL−1β is followed by better cognitive function 7 days after anesthesia
Chen et al (2019)	IL‐6	Inflammatory	Serum	Clinical	Patients developed into delirium had significant higher level of IL−6 at 6, 12 and 18 hours after surgery
Quan et al (2019)	CRP	Inflammatory	Plasma	Clinical	Lower CRP is followed by better cognitive function 7 days after anesthesia
Zhu et al (2020)	TNFα	Inflammatory	Plasma	Clinical	Higher level of TNFα is followed with lower MMSE score
Hov et al (2017)	S100β	Neurodegenerative	CSF	Clinical	In patients without preoperative delirium, higher S100β was observed in those develop into POD
Hassan et al (2020)	S100β	Neurodegenerative	Serum	Clinical	Patients without neuroprotective management had higher level of S100β followed with poorer cognitive performance 7 days after surgery.
Ballweg et al (2021)	Tau	Neurodegenerative	Plasma	Clinical	Plasma tau was significantly associated with delirium severity (*p* = 0.026) 1 day after surgery
Henjum et al (2018)	*Trem2*	Neurodegenerative	CSF	Clinical	Delirium was associated with higher levels of TREM2 in patients without pre‐existing dementia (*p* = 0.046)
Jiang et al (2018)	*Trem2*	Neurodegenerative	Hippocampus	Preclinical	The expression of *Trem2* gene was down regulated after surgical trauma on day 3, 7 and 14 followed with behavioral changes in Morris Water Maze
Passamonti et al (2019)	FC	Neuroimaging	Rs‐fMRI data PET data	Clinical	Neuroinflammation in AD induced abnormal FC
Franzmeier et al (2019)	FC	Neuroimaging	Rs‐fMRI data PET data	Clinical	FC from rs‐fMRI between any given region of interest (ROI) pair was associated with higher covariance in tau‐PET binding in the same ROIs
Mu et al (2020)	ALFF	Neuroimaging	Rs‐fMRI data	Clinical	AD patients without depression had higher increased ALFF on bilateral superior frontal gyrus, left middle frontal gyrus and left frontal gyrus
Zhuang et al (2020)	ALFF	Neuroimaging	Rs‐fMRI data	Clinical	MCI patients with aggregation vascular risk factors had different ALFF as compared with those without the risks.
Parisot, et al (2018)	Topology structure	Neuroimaging	Rs‐fMRI data	Clinical	The topology structures are different in AD and ASD patients

Note

AD, Alzheimer's disease; ALFF, amplitude of low frequency fluctuations; ASD, autism spectrum disorder; CRP, C‐reactive protein; CSF, cerebral spinal fluid; FC, functional connectivity; IL, interleukin; PET, positron emission tomography; rs‐fMRI, resting‐state functional magnetic resonance imaging; TNF, tumor necrosis factor; *Trem*, triggering receptor expressed on myeloid cell.

Commonly used inflammatory cytokines include IL‐1β,^112^ IL‐6,^113^ C‐reaction protein (CRP),^112^ TNFα,^114^ etc. Although these inflammatory cytokines have been proved to change significantly in preclinical and clinical settings, their specificity are rather low. For example, increased expression of peripheral IL‐1β is also associated with other conditions such as kidney disease and autoimmunity.^115^ In this case, postoperative elevation of IL‐1β should not be simply interpreted as a cognitive impairment predictor. Another example is that the increased expression of CRP inhibits lysis by recruiting factor H and activate complement C3b on damaged cells.^116^ However, the C3b is known for activating microglia,^117^ which may further induce PNDs. These results suggested that there is no single inflammatory cytokine that could accurate predict PNDs. Further investigations should focus on which combination of cytokine biomarkers would provide best sensitivity and specificity.

The cerebrospinal fluid (CSF) and serum/plasma biomarkers are largely used in neurodegenerative diseases. The rationale for utilizing these biomarkers is that the PNDs and neurodegenerative diseases share similar clinical symptoms, and that PND patients have higher risks for future neurodegenerative disorders. The S100β protein, tau protein, and *Trem2* are all well‐established CSF biomarkers in AD development.^118^ Recent studies found that both CSF and serum S100β levels were significantly higher in patients developed into PNDs as compared to those who did not.^119^, ^120^ The CSF *Trem2* is another promising biomarker for predicting neurodegenerative diseases.^121^ Both clinical and preclinical studies provided evidence for *Trem2* in PNDs.^67^, ^68^ Another observational study found that the change from preoperative to postoperative plasma tau protein level is associated with POD incidence and severity.^122^ A clinical difficulty in anesthesia is that the CSF is hard to obtain, limiting the use of CSF specimen. Meanwhile, whether the expressions of these proteins are responsible for inducing PNDs need further investigation, since a causal relationship between these proteins with PNDs after surgery and anesthesia has not been confirmed yet.

Advances in imaging technology have greatly broadened our scope in studying brain function. The positive emission tomography (PET) examination has been used to evaluate neuroinflammation for years.^123^ The resting‐state functional magnetic resonance imaging (rs‐MRI) is a recently developed non‐invasive method to study brain functions. Human brain functions as networks,^124^ and large amount of studies from recent years have elucidated various changes in functional connectivity (FC),^125^, ^126^ amplitude of low frequency fluctuations (ALFF)^127^, ^128^ and topology structures^129^ in patients with cognitive impairment. Recent studies aimed to utilize rs‐MRI to analyze cognitive function after surgery.^130^, ^131^ However, concerns are raised since the results are not replicable in different studies, and different post‐processing methods may result in different MRI analyzing results. Whether the rs‐fMRI or PET can be used for predicting PNDs still need further investigation.

## 5 POTENTIAL THERAPEUTIC STRATEGIES: IF WE CAN CURE OR PREVENT THE PNDS FROM ITS HAPPENING?

Although preclinical experiments pointed out several pathways that may be beneficial for clinical therapy, we must keep in mind that they are mainly used for preventing PNDs instead of curing the PNDs. As a result, the best PND management strategy is prevention from disease happening, through intraoperative anesthesia management for example, rather than treatment after disease onset.

### 5.1 Ulinastatin

Targeting NF‐κB signaling pathway is the closet to clinical practice since there are drugs available. Ulinastatin is a hydrolase protein inhibitor obtained from human urine. The ulinastatin was initially used for acute pancreatitis^132^ while has recently been used for intraoperative anti‐inflammatory management in elderly patients. Clinical data have shown that adding ulinastatin to aged patients is effective to improve mini‐mental state examination (MMSE) score after spinal surgery.^24^ In a meta‐analysis enrolling 10 high‐quality studies, ulinastatin treatment led to lower levels of inflammatory cytokines after surgery.^133^ Preclinical experiments confirmed that its anti‐inflammatory mechanisms involve blockade of the NF‐κB signaling pathway.^134^ Notably, some studies have reported a tendency of decreased mortality in patients following ulinastatin treatment.^132^, ^135^ Whether the treatment may also reduce the mortality in patients with PNDs still need further validation.

### 5.2 Dexmedetomidine

Dexmedetomidine is a highly selective alpha‐2 (α_2_) adrenoceptor agonist with sedative, analgesic, anxiolytic, sympatholytic, and opioid‐sparing properties.^136^ Using dexmedetomidine has been proved to be able to reduce the rate of PNDs both after surgery and in the ICU.^3^, ^31^, ^137^ Evidence from preclinical experiment found the protective effect may be the α_2_ receptor agonist activation.^138^ Meanwhile, after giving the dexmedetomidine, the NF‐κB signaling pathway activation was also decreased.^139^, ^140^ These results also suggested that the dexmedetomidine attenuated the inflammatory responses and improved cognitive function by reducing the activation of NF‐κB signaling pathway. However, most of the studies mainly focused on POD,^30^, ^31^, ^39^ the long‐term effect of dexmedetomidine on cognitive function still lacks enough evidence. Meanwhile, some of the studies draw contradictory results.^31^, ^39^ What is more, there are few studies focused on long‐term mortality in elderly patients. Thus, the effect of using dexmedetomidine still need further investigations.

### 5.3 Parecoxib sodium

Parecoxib sodium, a selective cyclooxygenase‐2 (COX‐2) inhibitor, is one of the most widely used non‐steroid anti‐inflammatory drugs (NSAIDs) in clinical practice. Recent studies have shown that parecoxib sodium resulted in decreased rate of PNDs,^141^, ^142^ possibly through its inhibiting the COX‐2 activity.^143^ However, there are several concerns regarding the use of parecoxib sodium in preventing/treating PNDs: (1) most of the studies focused on POD or delayed neurocognitive recovery. Its long‐term effect in cognitive impairment is unknown; (2) parecoxib sodium is not the only drug used in most studies. This may lead to confounding results. Whether parecoxib sodium is effective for preserving long‐term cognitive function after anesthesia and surgery needs further investigations.

## 6 CX3CL1/CX3CR1 AND *TREM 2* GENE EXPRESSION

Although evidence from clinical practice is lacking, preclinical experiment in aged rats suggested that blocking CX3CL1/CX3CR1 signaling with neutralizing antibody reduced inflammatory cytokine secretion and hippocampal astrocyte activation, and improved behavioral performances.^70^, ^144^ As for microglial *Trem2*, preclinical experiments found overexpression of *Trem2* could downregulate inflammatory cytokines secretion and improve behavioral performances in rats after surgery.^67^ Meanwhile, clinical data also suggested that CSF *Trem2* levels are highly associated with post‐surgery delirium in patients without pre‐existing dementia. However, direct evidence proving that regulating CX3CL1/CX3CR1 and *Trem2* expression can improve cognitive function after surgery is still lacking.

Although advances from both clinical practice and life science pointed out the potential causes and therapeutic targets for PNDs, it is still a major challenge for anesthesia practice. It is under debate whether the PNDs are highly related to cognitive impairment and other type of neurodegenerative disease later in life. Thanks to the sequencing method, the ACE gene missense mutation was found to be responsible for an early‐onset, rapid progressing dementia.^145^ This finding highlighted the significance of gene‐sequencing method to studying PND mechanisms. Meanwhile, it also pointed out a novel direction for mechanistic studies, aside from traditional approaches such as taking blood samples and doing neuropsychological assessment. Another study found that the activation of PKC signaling pathway enhanced the treatment effect in refractory depression,^146^ implying there might be different signaling pathways in different types of PNDs.

Aside from neurodegenerative disease, neurovascular disease may also cause cognitive dysfunction. For example, clinical practice shown that management of stroke risk factors may also reduce later dementia.^147^ Another evidence is that elderly patients with NOTCH3 cysteine altering variants have higher risks of both stroke and cognitive impairment.^148^ Meanwhile, brain dysfunction such as delirium can also be found in patients with stroke history.^149^ These findings implying potential directions for PND studies.

Although signaling pathways responsible for PND still need further investigation, signaling pathways relating to other neuropsychological diseases are highly valuable to refer to in PND studies. Since preclinical experiments showed that the mechanisms of PNDs may be similar to those of neurocognitive disorders,^150^ and PND patients share similar clinical symptoms with neurodegenerative disorders and neurovascular disorders in some extent, studies on the latter would reveal new directions for PND studies both preclinically and clinically.

Since the PNDs are hard to cure by far, future studies should focus on its etiology and risk factors to reduce its incidence. Meanwhile, preclinical experiments should focus on the long‐term activation of astrocytes and crosstalk among microglia, astrocytes and neurons. We believe that effort from both clinical and preclinical studies will finally benefit patient care.

## CONFLICT OF INTEREST

The authors declare no conflicts of interest.

## AUTHOR CONTRIBUTIONS

Yang Liu: writing the manuscript; Huiqun Fu: illustration drawing; Tianlong Wang: reviewing manuscript.

## Data Availability

This is a review article and all the references have been published online.
